# The effect of web-based educational intervention on psychological status and blood glucose in newly diagnosed patients with diabetes type 2 in rural China: A protocol for randomized trial

**DOI:** 10.1097/MD.0000000000024937

**Published:** 2021-02-26

**Authors:** Zhixiang Yuan, Nini Jiao, Xiaoli Liu, Changjiang Liu

**Affiliations:** a Department of Finance; b Department of Endocrinology, Chengyang People's Hospital, Qingdao, China.

**Keywords:** protocol, randomized, type 2 diabetes mellitus, web-based educational intervention

## Abstract

**Background:**

No studies were located which used a web-based educational intervention to improve the knowledge about newly diagnosed type 2 diabetes mellitus (T2DM). Therefore, the primary objective of the present study was to evaluate the efficacy of web-based educational intervention on psychological outcomes and glycemic control in newly diagnosed T2DM in rural China.

**Methods:**

This work is a part of a comprehensive research project to assess and provide educational intervention that potentially improve psychological status and blood glucose among patients with T2DM. Eligibility criteria for the study includes newly diagnosed with T2DM, adult patients (age ≥30 years) regardless of gender; speak and understand Chinese languages; having no significant comorbidity; being not involved in any trial/study related to diabetes during last 3 months and able to attend regular visits. Eligible participants were divided into 2 groups according to completely randomized design: education group and control group. The outcomes included fasting blood glucose level, EQ-5D-3L questionnaire, Self-rating Anxiety Scale, and Self-rating Depression Scale.

**Results:**

This protocol will provide a reliable theoretical basis for the following research.

**Conclusion:**

The sample came from a single health centre. Therefore, the results can not be generalized for the entire population.

**Trial registration:**

This study protocol was registered in Research Registry (researchregistry6511).

## 1 Introduction

Type 2 diabetes mellitus (T2DM) is becoming a threat to people's health all over the world, also in China. The overall prevalence of T2DM in China increased sharply from 0.7% to 9.7% between 1980 and 2010.^[1]^ The dramatic urbanization, the aging population, the rapid change of lifestyle, with increasing overweight and obesity are suggested as possible reasons for the rapid growth of T2DM. The situation regarding T2DM in rural China is even more challenging, with a faster rate of increase of incidence than in the urban areas, while the awareness, treatment, and control of diabetes are less adequate in rural areas.^[2–4]^ Costly hospital care remains dominant in diabetes care in rural areas. Poverty is still a major problem, and affordable and adequate health care is still not available for rural residents.

The primary care services in rural China consists of township health centers and village clinics, and almost all of them are publicly owned. Physicians and nurses in primary care often have low levels of training.^[5,6]^ The most fundamental functions of primary care institutions include implementing basic public health, including establishing health records for residents, health education, and management of chronic diseases. Much of the care of chronic diseases, however, is provided by hospitals and there is little integration between hospitals and primary care services.^[7]^

Nowadays, the internet has become a very popular source of health information for patients in China. Many patients utilize the Internet as a primary resource for information about their disease, and as a means to help them deal with concerns or navigate decisions related to T2DM and improve their knowledge of controlling blood glucose.^[8–10]^ This popularity may relate to advantages, such as immediate access, usually at home, which avoids travel and is cost saving. Patients with T2DM in rural are one such group of users. The present study introduced a web-based education program for patients with T2DM in rural which aimed to support T2DM education and self-management. The program was developed in close cooperation with potential users and is based on national guidelines for diabetes care.^[11]^

Finally, although a number of studies have reported on knowledge evaluation among patients with type 1 and gestational DM, literature related to knowledge evaluation among patients with newly diagnosed T2DM is limited, and no studies were located which used a web-based educational intervention to improve the knowledge about newly diagnosed T2DM. For these reasons, this study focused on evaluating patients of newly diagnosed T2DM in rural China, self-management, healthy diet, exercise, and lifestyle habits.^[12]^

Therefore, the primary objective of the present study was to evaluate the efficacy of web-based educational intervention on psychological outcomes and glycemic control in newly diagnosed T2DM in rural China.

## 2 Materials and methods

### 2.1 Ethical approval

This work is a part of a comprehensive research project to assess and provide educational intervention that potentially improve psychological status and blood glucose among patients with T2DM. This research project has been registered in the research registry (with number: researchregistry6511) and received ethical approval from the Medical Research and Ethics Committee in Chengyang People's Hospital (no. NMRR-20-903-40294). Also, before the participating in the educational outreach intervention, all health care providers were briefed about the study objectives and the voluntary basis of this participation. After the introductory briefing, the acceptance to take the presession questionnaire implied the participant's consent to participate in the study.

### 2.2 Study population

Eligibility criteria for the study includes newly diagnosed with T2DM, adult patients (age ≥30 years) regardless of gender; speak and understand Chinese languages; having no significant comorbidity; being not involved in any trial/study related to diabetes during last 3 months and able to attend regular visits. Participants will be excluded if they are of other types of diabetes (gestational diabetes, T1DM); unable to answer the questionnaire independently or having hearing, vision, or cognitive impairments.

### 2.3 Random allocation

**Figure 1:**
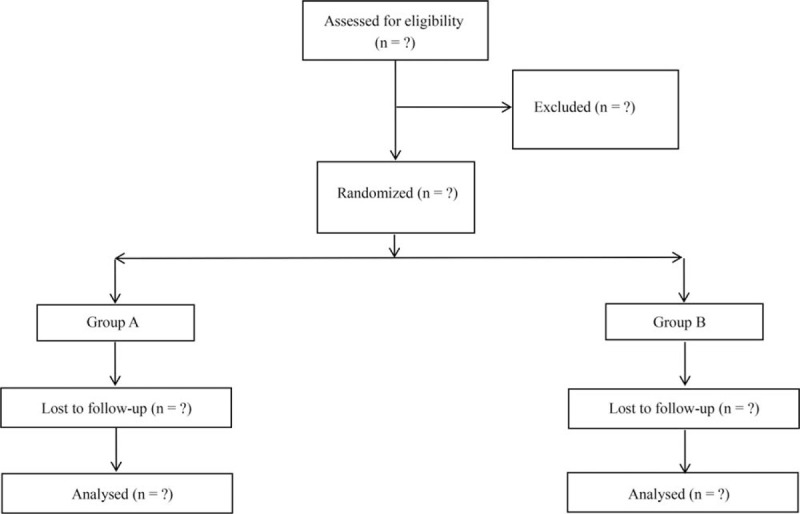
Consolidated Standards of Reporting Trials (CONSORT) diagram of patient flow through the study.

Eligible participants were divided into 2 groups according to completely randomized design: education group and control group (Fig. 1). The program duration was 6 months. The education of the patients was accomplished by professional education nurses. All nurses were well-trained.

### 2.4 Intervention and control protocols

Patients in the intervention group attended the standard clinic-based education and additionally used the web-based education intervention. Access to the web-based education intervention (A touch screen computer) took place at the diabetes clinic at our hospital, supervised by the researcher. The program provided information on healthy diet and healthy lifestyle and included pictures and simple instructions. The program took 15 to 30 minutes to complete. Patients were also given the URL link for the website, so they could access the website as they wished at home.

Patients in the control group attended the standard clinic-based education class alone. The single, group education class, lasting 1.5 hours, is run by Health's dieticians and diabetes educator nurses. Generally 5 to 8 newly diagnosed T2DM patients attend. Session and content is based on healthy diet, exercise, healthy lifestyle, and blood glucose level monitoring.

### 2.5 Outcomes

Questions on the patients’ sociodemographic characteristics concerned their age, sex, marital status, level of education, and occupation type (Table 1). Fasting blood glucose level was measured in mmol/L from a venous blood sample. Health-related quality of life was measured by the EQ-5D-3L questionnaire, which is a validated generic instrument used in clinical settings for general population and for economic evaluation. Based on the real-time information, we would also assess the patient conditions and offer corresponding suggestions for better self-management. The Self-rating Anxiety Scale and Self-rating Depression Scale are the scales for assessing anxiety and depression, which includes 20 problems respectively, using a 4-point scale ranging from 1 (none, or a little of the time) to 4 (most, or all of the time). The statistical score of all questions were calculated after completion of the answers (Table 2).

**Table 1:** Patient baseline demographics.

Demographics	Education group	Control group	*P* value
Number of patients			
Age^†^ (years)			
Male sex (no. [%])			
BMI^†^ (kg/m^2^)			
Follow-up^†^			

BMI = body mass index.

† The values are given as the mean and the SD.

**Table 2:** Postoperative outcomes.

Outcomes	Education group	Control group	*P* value
Fasting blood glucose level^†^			
EQ-5D-3L questionnaire^†^			
Self-rating Anxiety Scale^†^			
Self-rating Depression Scale^†^			

† The values are given as the mean and the SD.

### 2.6 Statistical analysis and power analysis

The analyses will be performed using SPSS 22.0.0 (SPSS Inc., Chicago, IL). Frequencies and descriptive statistics will be used for patients’ demographic presentation, while means and standard deviations will be calculated for the continuous variables and group differences will be analyzed by using Pearson chi-square test for categorical variables. Kolmogorov–Smirnov test will be applied to check the distribution of data. Independent t test will be used in case of normally distributed data, whereas, Mann–Whitney *U* test for non-normal distributions. *P* <0.05 will be considered as significant for all analysis.

## 3 Discussion

Although a number of studies have reported on knowledge evaluation among patients with type 1 and gestational DM, literature related to knowledge evaluation among patients with newly diagnosed T2DM is limited, and no studies were located which used a web-based educational intervention to improve the knowledge about newly diagnosed T2DM. For these reasons, this study focused on evaluating patients of newly diagnosed T2DM in rural China, self-management, healthy diet, exercise, and lifestyle habits. Therefore, the primary objective of the present study was to evaluate the efficacy of web-based educational intervention on psychological outcomes and glycemic control in newly diagnosed T2DM in rural China.

Among the limitations of the trial, it should be noted that no blinding was used regarding group allocation, which is difficult in this type of study. Secondly, changes in medication were not taken into account. Medication adjustments in these participants are inevitable, and they may have an influence both on the intervention and control group. Finally, the sample came from a single health center. Therefore, the results cannot be generalized for the entire population.

## Author contributions

**Conceptualization:** Zhixiang Yuan, Xiaoli Liu.

**Data curation:** Zhixiang Yuan.

**Formal analysis:** Zhixiang Yuan, Nini Jiao.

**Funding acquisition:** Changjiang Liu.

**Investigation:** Zhixiang Yuan, Nini Jiao.

**Methodology:** Xiaoli Liu.

**Project administration:** Changjiang Liu.

**Software:** Zhixiang Yuan, Xiaoli Liu.

**Validation:** Nini Jiao, Xiaoli Liu.

**Writing – original draft:** Zhixiang Yuan.

**Writing – review & editing:** Changjiang Liu.
